# Ideal cardiovascular health influences cardiovascular disease risk associated with high lipoprotein(a) levels and genotype: The EPIC-Norfolk prospective population study

**DOI:** 10.1016/j.atherosclerosis.2016.11.010

**Published:** 2017-01

**Authors:** Nicolas Perrot, Rutger Verbeek, Manjinder Sandhu, S. Matthijs Boekholdt, G. Kees Hovingh, Nicholas J. Wareham, Kay-Tee Khaw, Benoit J. Arsenault

**Affiliations:** a Centre de Recherche de l’Institut Universitaire de Cardiologie et de Pneumologie de Québec, Canada; b Department of Medicine, Faculty of Medicine, Université Laval, Québec, Canada; c Department of Vascular Medicine, Academic Medical Centre, Amsterdam, The Netherlands; d Department of Public Health and Primary Care, University of Cambridge, Cambridge, United Kingdom; e Genetic Epidemiology Group, Wellcome Trust Sanger Institute, Hinxton, United Kingdom; f Department of Cardiology, Academic Medical Centre, Amsterdam, The Netherlands; g MRC Epidemiology Unit, Cambridge, United Kingdom

**Keywords:** Lipoprotein(a), Lifestyle, Genetics, Ideal cardiovascular health, Cardiovascular diseases

## Abstract

**Background and aims:**

Lipoprotein(a) (Lp[a]) is a strong genetic risk factor for cardiovascular disease (CVD). The American Heart Association has prioritised seven cardiovascular health metrics to reduce the burden of CVD: body mass index, healthy diet, physical activity, smoking status, blood pressure, diabetes and cholesterol levels (together also known as ideal cardiovascular health). Our objective was to determine if individuals with high Lp(a) levels could derive cardiovascular benefits if characterized by ideal cardiovascular health.

**Methods:**

A total of 14,051 participants of the EPIC-Norfolk study were stratified according to the cardiovascular health score (based on the number of health metrics with an ideal, intermediate or poor status). Of them, 1732 had a CVD event during a mean follow-up of 11.5 years. Cox proportional hazards models were used to describe the association between the cardiovascular health score and Lp(a) level or genotype (as estimated by the rs10455872 variant) with the risk of CVD.

**Results:**

We observed little or no differences in serum Lp(a) levels across the seven cardiovascular health metric categories. Among participants with high serum Lp(a) levels ≥50 mg/dl), those in the highest (i.e. healthiest) cardiovascular health score category (10–14) had an adjusted hazard ratio for cardiovascular disease of 0.33 (95% CI = 0.17–0.63, *p* = 0.001) compared to participants in the lowest (i.e. unhealthiest) cardiovascular health score category(0–4). Similar results were obtained when we replaced Lp(a) with rs10455872.

**Conclusions:**

Although Lp(a) levels are only slightly influenced by cardiovascular health metrics, an ideal cardiovascular health could substantially reduce CVD risk associated with high Lp(a) levels or genotype.

## Highlights

• Elevated lipoprotein(a) levels is one of the strongest genetic risk factors for CVD.
• Few health professionals routinely measure Lp(a) presumably because there are no therapeutic agents available to lower Lp(a).
• Clinical and lifestyle-related factors do not appear to influence Lp(a) levels at the population level.
• Individuals with high Lp(a) levels who control clinical and lifestyle-related risk factors have a lower risk of CVD.
• Targeting clinical and lifestyle-related risk factors could reduce cardiovascular risk in individuals with high Lp(a) levels.

## 1 Introduction

Lipoprotein(a) [Lp(a]) consists of a cholesterol rich lipoprotein particle analogous to low-density lipoprotein (LDL), where apolipoprotein B-100 (the most abundant protein on LDL particles) is linked to apolipoprotein (apo)(a) by a disulphide bond. Individuals with Lp(a) levels in the top 20% of the population distribution (i.e. above approximately 50 mg/dL) have a 2–3 fold higher increased risk of coronary heart disease and stroke [1], [2]. In fact, recent studies suggest that Lp(a) could be one of the strongest genetic risk factor for cardiovascular disease (CVD) [3]. The *LPA* gene is highly polymorphic and one of the strongest determinants of Lp(a) levels is a copy number variation (CNV) at the *LPA* locus encoding the number of KIV-2 repeats [4]. The single nucleotide polymorphism (SNP) rs10455872 has been shown to be closely associated with KIV-2 repeats and Lp(a) levels [5], [6]. These genetic variations at the *LPA* locus have been suggested to strongly influence Lp(a) levels by altering its hepatic secretion rate [4]. Up to 90% of the variance in circulating Lp(a) could be explained by genetic factors [7]. Lp(a) is the preferential carrier of oxidized phospholipids in the blood [8], which may be the underlying reason for its atherogenic potential.

In 2010, a European Atherosclerosis Society Consensus Panel recommended that Lp(a) levels should be measured in individuals with premature CVD (or with a family history of premature CVD), familial hypercholesterolemia, recurrent CVD despite statin treatment or intermediate cardiovascular risk [1]. However, even in these populations, Lp(a) is currently not routinely measured. The lack of a consensus on a validated and standardized Lp(a) assay, our incomplete understanding of the atherogenecity of Lp(a) and relative unawareness of Lp(a) amongst health care providers probably are factors that could explain why Lp(a) is not routinely measured. Moreover, a clinically validated specific therapy against Lp(a) is not available.

The American Heart Association (AHA) 2020 Strategic Impact Goals introduced the concept of ideal cardiovascular health, which includes seven cardiovascular health metrics, namely body mass index, a healthy diet, physical activity, smoking status, blood pressure, fasting plasma glucose and cholesterol levels [9]. Over the past 5 years, several prospective studies have shown that compared to people who meet few of the criteria of ideal cardiovascular health, those with ideal cardiovascular health have a 80–90% lower risk of CVD events [10], [11], [12]. Whether individuals with high Lp(a) can reduce their risk of cardiovascular events by controlling other risk factors such as those of the AHA's ideal cardiovascular health is unknown. In this study, we hypothesised that Lp(a) levels will be comparable across the seven healthy lifestyle risk factors and that individuals with high Lp(a) levels (or carriers of G allele of the Lp(a)-raising SNP rs10555872) are at lower risk of cardiovascular events if they are characterized by ideal cardiovascular health. We tested these hypotheses in the European Prospective Investigation into Cancer and Nutrition (EPIC)-Norfolk study.

## 2 Materials and methods

### 2.1 Study design

The EPIC-Norfolk prospective population study is a population-based cohort of 25,639 men and women, aged between 39 and 79 years, resident in Norfolk, United Kingdom. The design and methods of the study have been described previously [13]. Participants were recruited from age-sex registers of general practices in Norfolk as part of the 10-country collaborative EPIC study. The study cohort was closely similar to UK population samples for many characteristics, including anthropometry, blood pressure and lipids, but with a lower proportion of smokers. At the baseline survey conducted between 1993 and 1997, participants completed a detailed health and lifestyle questionnaire. Blood was taken by venipuncture into plain and citrate tubes. Blood samples were processed for various assays at the Department of Clinical Biochemistry, University of Cambridge, or stored at −80 °C.

Participants were identified as having been hospitalised or having died because of a cardiovascular event if the corresponding International Classification of Disease (ICD)-10 code was recorded as the underlying cause of hospitalisation or mortality. Hospitalised participants were identified using their unique National Health Service number linked with the East Norfolk Health Authority (ENCORE) database. The ENCORE database identified all hospital contacts throughout England and Wales for residents of Norfolk. Death certificates were coded by trained nosologists according to ICD-10. Deaths or hospitalisations were attributed to coronary heart disease (CHD) if the underlying cause was coded by as ICD-10 codes 120–125, which encompass the clinical spectrum of CHD, including unstable angina, stable angina and myocardial infarction. Deaths or hospitalisations were attributed to stroke if the underlying cause was coded as ischaemic (I63, I65, I66) or haemorrhagic stroke (I60–62). CVD was defined as either a CHD or stroke. The follow-up was censored on 31 March 2008. The study protocol was approved by the Norwich District Health Authority Ethics Committee and all participants gave written informed consent.

### 2.2 Definition of cardiovascular health metrics

Ideal cardiovascular health metrics were classified as ideal, intermediate or poor according the seven risk factors identified by the AHA, as previously described [12]. Body mass index (BMI) was classified as ideal if < 25 kg/m^2^, as intermediate if 25–30 kg/m^2^ or as poor if ≥ 30 kg/m^2^. A healthy diet score (HDS) was based on intake of five dietary components. The first component was the intake of sufficient amounts of fruit and vegetables; a consumption of 4.5 or more cups per day was classified as meeting the guidelines. Second, the weight of estimated daily fish consumption was multiplied by seven and divided by 3.5 oz (portion size); if the value was ≥2, the participant was considered to consume two or more servings per week. Third, for fibre-rich whole grains, participants consuming three or more servings per day of 1 oz each were considered to meet the guidelines. The fourth and fifth HDS components were low sodium intake (<1500 mg per day was classified as healthy) and low consumption of sugar-sweetened beverages (<450 kcal per week was classified as healthy). The HDS was calculated as the sum of the number of healthy food items, yielding a HDS range of 0–5. HDS was categorised as ideal (≥4), intermediate (2–3), or poor (<2). Physical activity was defined as ideal, intermediate and poor if the status was active, moderately active or moderately inactive, and inactive, respectively, as previously described [14]. Smoking status was classified as ideal, intermediate or poor if the study participant had never smoked, previously smoked, or was a current smoker, respectively. Blood pressure was defined as ideal if systolic pressure was <120 mmHg and diastolic pressure was <80 mmHg, as intermediate if systolic pressure was 120–139 mmHg or diastolic pressure was 80–89 mmHg without antihypertensive drug treatment or if the blood pressure was at goal on treatment, or poor if systolic pressure was ≥140 mmHg or diastolic pressure was ≥90 mmHg. Total cholesterol levels were classified as ideal (<5.2 mmol/L), intermediate (5.2–6.2 mmol/L) or when cholesterol levels were treated to goal, or poor (≥6.2 mmol/L). Diabetes mellitus status was ascertained by means of (1) self-report of diabetes medication use or (2) a Hba1c ≥ 6.5 mmol/L. Participants meeting one of these criteria were attributed a value of 0 while individuals without diabetes mellitus were attributed a value of 2.

The overall cardiovascular health score (CHS) was calculated based on these seven health metrics, giving 2 points for an ideal metric, 1 point for an intermediate metric, and 0 points for a poor metric, thus yielding an overall CHS between 0 and 14. The CHS was divided into three categories as follows: 0–4 (unhealthy), 5–9 (intermediate) and 10–14 (healthy).

### 2.3 Genotyping and laboratory measurements

The rs10455872 genetic variant was genotyped using Custom TaqMan^®^ SNP Genotyping Assays (Applied Biosystems, Warrington, UK). The genotyping assays were carried out using 10 ng of genomic DNA in a 2.5 μl reaction volume on 384-well plates using a G-Storm GS4 Thermal Cycler (GRI, Rayne, UK). The ABI PRISM^®^ 7900HT Sequence Detection System (Applied Biosystems, Warrington, UK) was used for end-point detection and allele calling. The SNP passed the quality control criteria in the EPIC-Norfolk study (call rate > 95%, blind duplicate concordance ≥ 95%). Various laboratory measurements including a conventional lipid profile, were performed at baseline as previously described [13]. When additional funding became available in 2010, additional measurements were performed in a subset of the cohort with available stored frozen blood samples. Lp(a) levels were measured with an immunoturbidimetric assay using polyclonal antibodies directed against epitopes in apolipoprotein(a) (Denka Seiken, Coventry, United Kingdom), as previously described [15]. This assay has been shown to be apolipoprotein(a) isoform-independent.

### 2.4 Statistical analyses

Baseline characteristics of study participants were compared between participants with high *vs.* low Lp(a) levels using an unpaired Student t-test for continuous variables with a normal distribution or a chi-square test for categorical variables. Differences in median Lp(a) levels across cardiovascular health metrics with the exception of diabetes mellitus were tested using Kruskal Wallis one-way analysis. For the difference in median Lp(a) levels across the diabetes mellitus group, a Mann-Whitney *U* test was performed. The differences in percentages of participants with high Lp(a) levels (>50 mg/dL) and rs10455872 carriers across categories of ideal cardiovascular health metrics was tested using chi-square analysis. Cox proportional hazards models were used to calculate hazard ratios (HR) and corresponding 95% confidence interval (95% CI) for the risk of future CHD in participants separated on the basis of cardiovascular health metrics and Lp(a) levels (or genotype). Regression models were tested before and after adjustment for potential confounding risk factors such as age and sex. The interaction terms between Lp(a) and cardiovascular health *and* rs10455872 and cardiovascular health were calculated with the use of Cox proportional hazard models. Statistical analyses were performed using SPSS software version 20.

## 3 Results

A total of 14,051 participants with a complete data set available for ideal cardiovascular health metrics and Lp(a) levels were included in this analysis. Of them, 1732 had a CVD event during a mean follow-up of 11.5 years. The baseline characteristics of the study participants are presented in Table 1 for the entire study sample separated on the basis of Lp(a) levels (< or ≥50 mg/dL). There were no sex and age differences between participants with high *vs.* low Lp(a) levels. Body mass index, blood pressure, the percentage of participants with diabetes mellitus or smokers was also comparable between participants with high *vs.* low Lp(a). Cholesterol levels were higher in participants with high Lp(a). With the exception of sodium intake, all other intake parameters were not different in patients with high *vs.* low Lp(a) for healthy diet components or physical activity habits.

Table 2 presents median Lp(a) levels in patients with ideal, intermediate or poor cardiovascular health metrics. Lp(a) levels were significantly different across diet and physical activity categories but these differences were very small and the percentage of participants with Lp(a) levels ≥50 mg/dL or carriers of the G allele of rs10455872 was not different across physical activity and diet categories. As anticipated, cholesterol levels were different across Lp(a) categories, most likely because Lp(a) carries cholesterol. Consequently, Lp(a) levels appeared to be slightly influenced by the presence/absence of the cardiovascular health score.

**Fig. 1:**
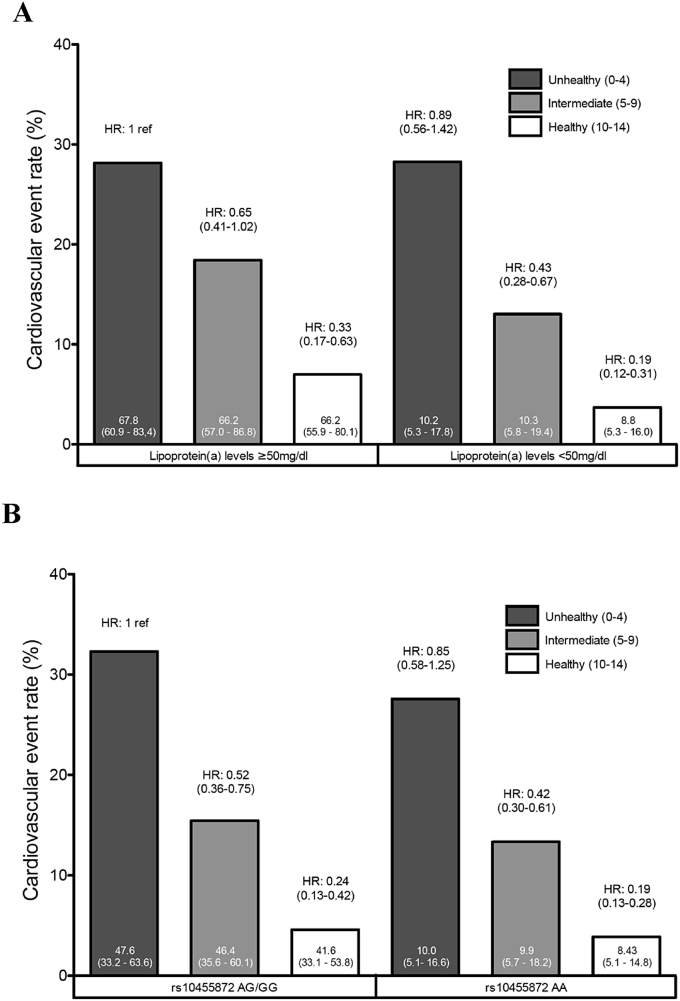
Cardiovascular disease event rates (bars) and hazard ratios for incident cardiovascular disease. In patients classified on the basis of ideal cardiovascular health categories and Lp(a) levels (A) and rs10455872 carrier status (B) after adjusting for age and sex. Median (interquartile range) Lp(a) levels are also presented in each categories.

The associations between the cardiovascular health score, Lp(a) levels and cardiovascular outcomes are presented in Fig. 1. Fig. 1A shows that among patients with high Lp(a), those with ideal cardiovascular health have a relative risk of CVD of 0.33 (95% CI, 0.17–0.63, *p* < 0.001) compared to those with poor cardiovascular health. Our results also show that patients with ideal cardiovascular health and low Lp(a) levels are those with the lowest CVD event rate (hazard ratio = 0.19, 95% CI, 0.12–0.31, *p* < 0.001). Similar results were obtained when we replaced Lp(a) with the Lp(a)-raising allele (G allele of rs10455872). Fig. 1B shows that among study participants with at least one Lp(a)-raising allele, those with ideal cardiovascular health have a relative risk of CVD of 0.24 (95% CI, 0.13–0.42, *p* < 0.001) compared to those with poor cardiovascular health. Our results also show that patients with ideal cardiovascular health who did not have the Lp(a)-raising allele are those with the lowest CVD event rate (hazard ratio = 0.19, 95% CI, 0.13–0.28, *p* < 0.001). We also computed the interaction terms between Lp(a) levels (or genotype) and health categories for CVD risk prediction and found that none were significant (data not shown), which suggests that there is no evidence that the relationship between health categories and CVD risk is affected by Lp(a) levels (or genotype).

## 4 Discussion

Family-based studies and genetic association studies conducted in the general population have consistently shown that the inter-individual variation in Lp(a) levels are most likely explained by genetic factors. In this study, we found that the seven AHA metrics of ideal cardiovascular health merely influence Lp(a) levels, which further supports the notion that lifestyle may not represent a key a factor in the management of high Lp(a) levels. However, the results of our study do suggest that among people with high Lp(a) levels, the management of lifestyle-related factors may reduce cardiovascular risk by up to 75%.

Cross-sectional and intervention studies have been performed to address the impact of physical activity levels and exercise training on Lp(a) levels. Mora et al. found no association between physical activity levels and Lp(a) levels in the Women's Health Study [16] This is in line with the outcomes of an intervention study where nine-months of aerobic training in 30 healthy adults was shown not to have an impact on Lp(a) levels. In patients with type 2 diabetes, resistance training resulted in a decrease in Lp(a) levels (from 15.4 ± 18 mg/dL to 13.8 ± 18 mg/dL, *p* = 0.04) [17], but another study found no such significant effect [18]. In our study, a healthy dietary pattern was not strongly associated with Lp(a) levels. Dietary intervention studies have, however, suggested that the macronutrient composition of the diet may influence Lp(a) levels. For instance, in a randomized crossover study, Faghihnia et al. [19] have shown that a 4-week low-fat, high-carbohydrate diet increased Lp(a) levels compared to a high-fat, low-carbohydrate diet in 63 healthy participants. In the Omni Heart trial, Haring et al. [20] compared the impact of 3 diets (high-carbohydrate, high-protein and high in unsaturated fats) on Lp(a) levels. Although changes in Lp(a) were modest (2,1 to 4,7 mg/dL), all diets significantly increased Lp(a) levels, with marked heterogeneity among black and white participants. Investigators who have measured Lp(a) levels in nutritional intervention studies aiming at reducing LDL cholesterol levels (with portfolio diet, almonds, flaxseed, etc.) also found little benefits of these interventions with regards to Lp(a) levels [21], [22], [23]. Drugs targeting LDL cholesterol levels such as statins also have little or no effect on Lp(a) levels. In fact, whereas most studies showed that statins do not reduce Lp(a) levels, some studies show that statins could even increase Lp(a) and oxPL levels [24], [25], [26]. Whether people with high Lp(a) levels would derive more benefits from statin therapy than people with lower Lp(a) levels is unknown.

Our study has limitations. For instance, our study population almost exclusively included Caucasians. Therefore, our results may not be applicable to other populations. Although we provide results from a large-scale prospective study with a follow-up of 11.5 years, the true impact of encouraging people (with or without elevated Lp(a) levels) to improve cardiovascular health by meeting the AHA criteria on CVD outcomes can only be formally tested in a randomized clinical trial. However, regardless of Lp(a) levels, there are currently no randomized clinical trials that have documented the potential benefits of meeting the AHA's criteria for ideal cardiovascular health.

The association between Lp(a) levels and CVD risk is strong and consistent across sexes and ethnicities. The measurement of Lp(a) is clinically useful as it enhances cardiovascular risk reclassification and discrimination, as recently observed in the Bruneck study and the Copenhagen City Heart Studies [27], [28]. To the best of our knowledge, our study is the first to document the benefits of ideal cardiovascular health in patients with high Lp(a). Previous analyses within the EPIC-Norfolk dataset have confirmed that high Lp(a) levels are significantly associated with future risk of peripheral arterial disease, coronary artery disease and aortic stenosis [5], [15], a finding that underscores the need for compounds that specifically target Lp(a) (reviewed in Ref. [29]) as they could provide substantial cardiovascular benefits to patients with high Lp(a) if properly tested in cardiovascular outcomes trials. However, our results suggest that if Lp(a)-lowering therapies should become available, these should be added on top of lifestyle management and on top of other agents that target risk factors for CVD such as LDL cholesterol and blood pressure in people who cannot manage these risk factors with lifestyle alone.

It is commonly accepted that one of the barriers to the routine measurement of Lp(a) levels in clinics is the absence of Lp(a)-lowering therapy. Although randomized clinical trials will ultimately be required to formally test this hypothesis, our results suggest that the management of seven other CVD risk factors could reduce CVD risk in patients with high Lp(a). We believe that our results should encourage health professionals to measure Lp(a) levels at least once in their patients and more routinely in patients treated with lipid-lowering therapy as well as other lifestyle-related risk factors to properly assess cardiovascular risk. In conclusion, our results suggest that controlling cardiovascular risk factors and prescribing physical activity and a healthy diet should be pivotal for the management of patients with high Lp(a).

## Conflict of interest

The authors declared they do not have anything to disclose regarding conflict of interest with respect to this manuscript.

## Financial support

EPIC-Norfolk is supported by program grants from the Medical Research Council UK and Cancer Research UK and with additional support from the European Union, Stroke Association, British Heart Foundation, and Research into Ageing. RV is supported by a grant from the European Union [TransCard: FP7-603091-2]. BJA holds a junior scholar award from the *Fonds de recherche du Québec: Santé (FRQS)*.

## Author contributions

NP designed the statistical analysis plan, interpreted the data and wrote the manuscript. RV performed statistical analyses, interpreted the data and reviewed the manuscript. MS coordinated the genotyping and reviewed the manuscript. SMB performed statistical analyses, interpreted the data and reviewed the manuscript. GKH interpreted the data and reviewed the manuscript. NJW coordinated the study and reviewed the manuscript. KTK coordinated the study and reviewed the manuscript. BJA designed the statistical analysis plan, interpreted the data and wrote the manuscript.

## Figures and Tables

**Table 1:** Baseline clinical characteristics of the total study population and the study population separated on the basis of high *vs*. low lipoprotein(a) levels.

	Total	Lp(a) levels mg/dl		*p* value
		<50	≥50	
	(N = 14,051)	(N = 12,511)	(N = 1540)	
Age, years	59.0 (±9.1)	58.9 (±9.1)	59.6 (±8.8)	0.002
Male, %	47.2 (7167)	47.6 (5955)	43.7 (673)	0.004
Body mass index, kg/m^2^	26.2 (±3.7)	26.2 (±3.7)	26.1 (±3.7)	0.604
Systolic blood pressure, mmHg	135.2 (±18.1)	135.2 (±18.1)	135.0 (±18.0)	0.694
Diastolic blood pressure, mmHg	82.4 (±11.1)	82.4 (±11.1)	82.3 (±10.9)	0.629
Diabetes, %	3.1 (438)	3.1 (390)	3.1 (48)	0.999
Total cholesterol, mmol/L	6.2 (±1.2)	6.1 (±1.2)	6.6 (±1.1)	<0.001
LDL cholesterol, mmol/L	4.0 (±1.0)	3.9 (±1.0)	4.4 (±1.0)	<0.001
HDL cholesterol, mmol/L	1.4 (±0.4)	1.4 (±0.4)	1.5 (±0.4)	<0.001
Triglycerides, mmol/L	1.8 (±1.1)	1.8 (±1.1)	1.8 (±0.9)	0.082
Healthy diet				
Fruit and vegetables ≥4.5 cups per day	74.2% (10425)	74.1% (9266)	75.3% (1159)	0.311
Fish ≥2 servings per week	47.9% (6726)	47.8% (5984)	48.2% (742)	0.794
Fiber-rich whole grains ≥3 servings per day	44.5% (6253)	44.5% (5567)	44.5% (686)	0.971
Sodium <1500 mg per day	3.6% (507)	3.5% (434)	4.7% (73)	0.012
Sugar-sweetened beverages ≤450 kcal per week	42.6% (5988)	42.7% (5337)	42.3% (651)	0.773
Physical activity				
Inactive	29.3% (4123)	29.1% (3646)	31.0% (477)	0.47
Moderately inactive	28.4% (3993)	28.5% (3569)	27.5% (424)	
Moderately active	23.1% (3242)	23.2% (2899)	22.3% (343)	
Active	19.2% (2693)	19.2% (2397)	19.2% (296)	
Smoking behaviour				
Current	11.5% (1615)	11.4% (1429)	12.1% (186)	0.73
Former	41.7% (5859)	41.8% (5225)	41.2% (634)	
Never	46.8% (6577)	46.8% (5857)	46.8% (720)	

**Table 2:** Median Lp(a) level and the percentage of study participants with high Lp(a) levels or carriers of the rs10455872 allele in each category of cardiovascular health metric.

Cardiovascular health metrics	Number	Median Lp(a) level [IQR]	Lp(a) ≥50 mg/dL	rs10455872 G allele carriers
		(N = 14,051)	(N = 1540)	(N = 2100)
Body mass index				
Ideal	5748	11.3 (6.1–27.2)	11.3% (651)	15.1% (867)
Intermediate	6398	11.5 (6.3–26.5)	10.8% (689)	14.9% (955)
Poor	1905	11.3 (5.7–25.1)	10.5% (200)	14.6% (278)
*p*-Value		0.27	0.49	0.87
Healthy diet score				
Ideal	1373	11.2 (6.1–25.1)	10.9% (149)	14.2% (195)
Intermediate	8741	11.7 (6.3–27.3)	11.2% (997)	15.4% (1342)
Poor	3937	11.0 (5.58–25.5)	10.5% (419)	14.3% (563)
*p*-Value		0.004	0.54	0.22
Physical activity				
Ideal	2693	11.2 (6.1–27.1)	11.0% (296)	15.4% (414)
Intermediate	7235	11.2 (6.0–25.8)	10.6% (767)	14.6% (1052)
Poor	4123	11.8 (6.4–27.9)	11.6% (477)	15.3% (632)
*p*-Value		0.018	0.28	0.43
Smoking behaviour				
Ideal	6577	11.5 (6.1–27.5)	10.9% (720)	14.9% (982)
Intermediate	5859	11.3 (6.1–26.0)	10.8% (634)	14.8% (866)
Poor	1615	11.4 (6.3–27.9)	11.5% (186)	15.6% (252)
*p*-Value		0.64	0.73	0.71
Blood pressure				
Ideal	2462	10.7 (6.1–25.9)	11.0% (270)	14.3% (352)
Intermediate	5979	11.3 (5.9–26.9)	10.8% (647)	15.3% (912)
Poor	5610	11.8 (6.3–26.7)	11.1% (623)	14.9% (836)
*p*-Value		0.029	0.89	0.53
Diabetes Mellitus				
Yes	428	11.2 (5.9–26.5)	11.0% (48)	15.5% (68)
No	13,613	11.4 (6.1–26.7)	11.0% (1492)	14.9% (2032)
*p*-Value		0.43	0.99	0.73
Total cholesterol				
Ideal	2630	8.7 (5.1–16.8)	4.5% (118)	12.0% (315)
Intermediate	5284	10.7 (5.9–25.0)	9.3% (492)	15.7% (828)
Poor	6137	13.8 (7.1–33.4)	15.2% (930)	15.6% (957)
*p*-Value		<0.001	<0.001	<0.001
Cardiovascular Health Score				
Ideal	2893	9.8 (5.6–20.8)	8.4% (243)	13.5% (393)
Intermediate	10,525	11.8 (6.4–28.1)	11.6% (1226)	15.3% (1612)
Poor	633	11.7 (5.8–24.1)	11.2% (71)	15.2% (96)
*p*-Value		<0.001	<0.001	0.06
